# Semi-Empirical Prediction of Turned Surface Residual Stress for Inconel 718 Grounded in Experiments and Finite Element Simulations

**DOI:** 10.3390/ma14143937

**Published:** 2021-07-14

**Authors:** Huachen Peng, Wencheng Tang, Yan Xing, Xin Zhou

**Affiliations:** 1 School of Mechanical Engineering, Jiulong Lake Campus, Southeast University, Nanjing 211189, China; jsszphuach1992@163.com; 2 Shenyang Liming Aero-Engine (Group) Ltd., Shenyang 110862, China; zhou525157569xin@126.com

**Keywords:** semi-empirical prediction, surface residual stress, Inconel 718, turning, finite element model

## Abstract

The surface residual stress after machining, especially for finishing, has a vital influence on the shape stability and fatigue life of components. The current study focuses on proposing an original empirical equation to predict turned surface residual stress for Inconel 718 material, taking tool parameters into consideration. The tool cutting-edge angle, rake angle, and inclination angle are introduced for the first time in the equation based on the Inconel 718 material turning experiments and finite element simulations. In this study, the reliability of simulation parameters’ setting is firstly calibrated by comparing the residual stresses and chips of the experiments and simulations. The changing trends of turned forces, temperatures of lathe tool nose, and surface residual stress with turning parameters are analyzed. Then, the predictive equation of surface residual stress is proposed considering relationships between the back-rake angle, the side-rake angle, and the tool cutting-edge angle, rake angle, and inclination angle. Moreover, the genetic algorithm optimizes the objective function to obtain the undetermined coefficients in the prediction equation. Finally, the predicted accuracy of the surface residual stress is proven by comparing the experimental, simulation, and prediction values. The results indicate that the predictive equation of surface residual stress has a good accuracy in predicting turned surface residual stress for Inconel 718 materials. The correlation coefficient, R, and absolute average error between the predicted and the simulated value are 0.9624 and 13.40%, respectively. In the range of cutting parameters studied and experimental errors, this study provides an accurate predictive equation of turned surface residual stress for Inconel 718 materials.

## 1. Introduction

Nickel-based superalloys are widely applied in the aviation industry, such as in the manufacture of aeroengines, because they have excellent mechanical performance under high temperature and pressure. Inconel 718 occupies more than half the proportion of the nickel alloys applied by major aeroengine manufacturers. Its stable mechanical properties at high temperature make it one of the important materials in the aviation industry [1,2,3,4]. Nonetheless, Inconel 718 is not only conducive to processing just for the ability to hold good high-temperature resistance mechanical performance. The cutting process produces vast heat on the machined surface of the components introduced by serious friction between tool, chip, and cut surface. Meanwhile, the heat resulting from the extrusion and sliding deformation of material is generated in the first shear zone. The material also adheres to the tool nose region as the cutting proceeds, further intensifying the heat accumulation in the tool nose region for deformation and friction [5]. This causes complex residual stress in the superficial layer of the component, which affects the stability of size of the components, especially for the thin-walled components. In particular, the tensile surface residual stress tends towards accelerating the growth of microcracks, shortening the service life of the parts [6,7]. Therefore, the surface residual stress plays a critical role in dimensional accuracy and working life of machined parts. Furthermore, in terms of machining Inconel 718, it is vital to study the prediction model of surface residual stress for controlling deformation and improving the working life of parts.

The machined surface residual stress for Inconel 718 is related to the parameters in machining and the parameters of lathe tools, including geometry parameters, coating of lathe tools, and wear condition in the machining process. The experiments and simulations about the surface residual stress for machining Inconel 718 have been carried out in many previous studies. The changes of residual stress with cutting parameters are affected by work hardening rate and yield strength under the action of initial load [8,9]. Meanwhile, the change trends of residual stress are different with rising feed, depth of cut, and cutting speed [10,11,12].

In the works of Madariaga et al. [8,9], the tensile residual stress remained stable under initial static load, but increased when it was close to the yield stress. The variation of the surface residual stress rested with the work-hardening rate and the yield strength of the surface and subsurface layer under the initial quasi-static load. Their other research [10] pointed out that when the feed rate climbed, the surface residual stress rose with the increasing cutting speed (the range of 30~70 m/min) or feed rate (the range of 0.15~0.25 mm/rev) in the feed direction. Hua and Liu [11] investigated the principal residual stress and fatigue properties of turning Inconel 718. Their research showed that the principal and surface residual stresses both increased with the growing feed rate (0.075~0.15 mm/rev). In da Silva’s study [12], a model of an orthogonal turning was founded to forecast the surface integrity for machining Inconel 718, and according to the model put forward by Wu [13] and Denguir [14], the change of the residual stress with cutting parameters was analyzed. The residual stress of material near the surface decreased slightly when the cutting speed rose, yet the surface residual stress climbed as the cutting depth grew.

In addition to the above research on the relationship between residual stresses and cutting parameters, the residual stresses affected by cutting tools have also been studied in recent years [2,12,15,16,17]. In the study of Sharman et al. [2], the increase of tool nose radius resulted in more distinct surface tensile residual stress and made the tensile stress layer deeper. The worn tool caused large tensile stress layers in machining shallow areas near the surface. The authors of [12] also revealed that the surface residual stress caused by the tool with negative rake angle was higher than that of the tool with positive rake angle. The coupled Euler–Lagrangian method was employed to simulate the residual stress resulting from tool geometry for Inconel 718 orthogonal cutting in [15]. The negative rake angle tended to form the compressive residual stress near the turned surface, as did the sharp cutting-edge radius. Moreover, ceramic-coated tools tended to remain in a large surface tensile stress state for machining Inconel 718 materials. Holmberg et al. [16] compared the residual stress levels of the sharp and blunt ceramic or carbide-tipped tools for milling Inconel 718. For the ceramic tool, the surface residual stress along the feed direction was lower than that along the perpendicular direction, and the surface residual stresses in both directions caused by the sharp tool were higher than that resulting from the worn tool. For the carbide-tipped tool, it was lower than that of the ceramic tool along both feed and perpendicular directions, but the surface residual stress caused by tool wear was higher than that of the sharp tool in the feed direction. In Madariaga’s study [17], the residual stress on the turned surface first increased to a threshold value and then decreased with growing flank wear along the turning direction.

In the research of Gupta et al. [18], the cutting forces of turning Inconel 800 alloy using minimum quantity lubrication decreased when the cutting speed and cutting tool angle rose and the feed rate declined. Furthermore, the tool wear became more severe as the cutting speed rose and therefore as feed grew. In our previous study [19], Lorentz function and random forest regression were used to predict the distribution of residual stress in the depth direction, which did not introduce the tool angles. Ma et al. [20] proposed a milling surface residual stress prediction model, including milling parameters, milling force, and milling temperature, after analyzing forces and temperatures during milling Inconel 718 under the varying feeds and cutting depths.

In summary, there are few studies on the prediction model, including tool parameters, cutting parameters, cutting forces, and cutting temperature, in terms of surface residual stress for turning Inconel 718 material. Therefore, this current study concentrates on developing an innovative turned surface residual stress prediction model for Inconel 718, (1) containing the tool parameters, (2) the turning parameters, (3) turning forces, and (4) temperature, based on the experiments and simulations. In this prediction model, tool parameters are introduced for the first time. Finally, comparing predicted, simulated, and measured results, the effectiveness of the prediction model in the cutting parameters studied in this paper is well-validated.

## 2. Methods

### 2.1. Three-Dimensional FEM Simulation

**Figure 1:**
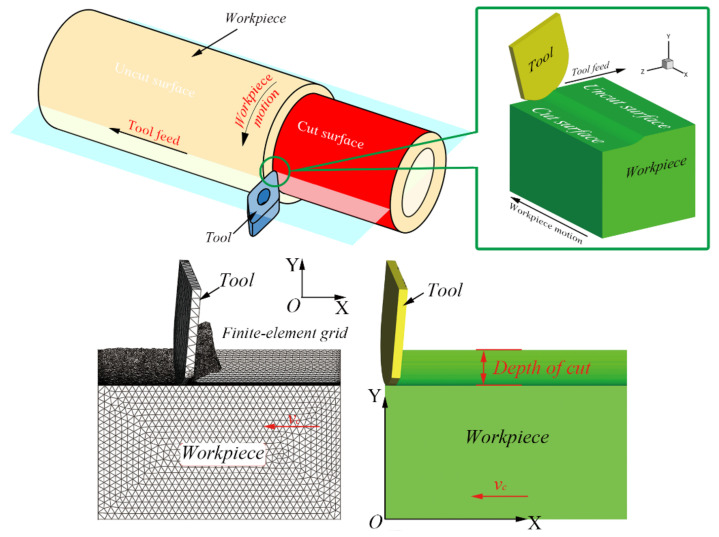
The 3D cylindrical turning model and boundary conditions [21].

In terms of analyzing the residual stresses near turned surface for Inconel 718 materials, it is necessary to establish a three-dimensional (3D) finite element turning model. The tool nose area can be approximately regarded as a plane for larger diameter cylindrical turning. Therefore, a 3D finite element turning model was established considering the region near the tool nose, using AdvantEdge software (V7.4015, Third Wave Systems, Minneapolis, MN, USA). The length, width, and height remain at 5 × 3 × 2 mm in the turning model. The tetrahedral elements were applied with the upper and lower limits of 0.15 and 0.005 mm element sizes. Then, the software automatically divided the mesh and kept more precise mesh generation in the cutting area. As displayed in Figure 1, the same as the movement form of cylindrical turning, the workpiece moves along the opposite direction of the X-axis with speed ***v_c_***, relative to the fixed tool in the simulation.

The Johnson-Cook constitutive model proposed in [22] was utilized for the simulation setup of the turning Inconel 718 material, which was expressed as Equation (1):

(1) $$\bar{\sigma }=\underset{Strain\;hardening}{\underbrace{\left(A+B{\bar{\varepsilon }}^{n}\right)}}\underset{Strain\;rate\;hardening}{\underbrace{\left(1+C\left(\frac{\dot{\bar{\varepsilon }}}{{\dot{\bar{\varepsilon }}}_{0}}\right)\right)}}\underset{Thermal\;softening}{\underbrace{\left(1-{\left(\frac{T-T_{room}}{T_{melt}-T_{room}}\right)}^{m}\right)}}$$

where $\bar{\sigma }$ denotes the equivalent flow stress (MPa). The equation includes three factors: (1) strain hardening term, (2) strain rate hardening term, and (3) thermal softening term. The strain hardening term involves yield strength (A), strain hardening coefficient (B), equivalent plastic strain ($\bar{\varepsilon }$), and strain hardening exponent (n). Additionally, the strain rate hardening term relates to strain rate hardening coefficient (C), equivalent plastic strain rate ($\dot{\bar{\varepsilon }}$), and reference equivalent plastic strain rate (${\dot{\bar{\varepsilon }}}_{0}$). Besides, the thermal softening term contains temperature (T), room temperature ($T_{room}$, with 20 °C), melting temperature ($T_{melt}$), and thermal softening exponent (m). In the present study, five parameters [23]: *A*, *B*, *C*, *m*, and *n*, as well as ${\dot{\bar{\varepsilon }}}_{0}$, were taken as 1290 MPa, 895 MPa, 0.016, 1.55, 0.526, and 0.03 $s^{-1}$, respectively [24]. The density of Inconel 718 is 8240 kg/m^3^ and the Young’s modulus and Poisson’s ratio are 214.58 GPa and 0.305, obtained separately in the X-ray residual stress measurement. The other material properties of Inconel 718 used in the simulation are shown in Table 1, and were obtained by X-ray diffractometer. The thermal conductivity varies at different temperatures, and so does the specific heat. Moreover, the coefficient of thermal expansion varies in different temperature ranges.

The D-type insert (55° top angle, grade: Carbide-Grade-M) was employed in the turning simulation as a rigid body, keeping nose radius 1.2 mm and edge radius 0.02 mm. Table 2 shows other tool geometrical parameters in turning simulations and experiments. The friction between insert and workpiece adopted the coulomb friction model and the coefficient was set at 0.23, and the tool elements adopted the tetrahedral mesh automatic generation technology in the AdvantEdge software, keeping the upper and lower limits of element sizes twice as large as those of the workpiece. Then, the software automatically generated the mesh and made it finer in the contact region between the insert and component. During the simulation, the workpiece moved 6 mm along the negative x-direction displayed in Figure 1, including 1 mm empty cutting, so that the chip completely separated from the workpiece.

### 2.2. Turning Experiments and Measuring Residual Stresses

**Figure 2:**
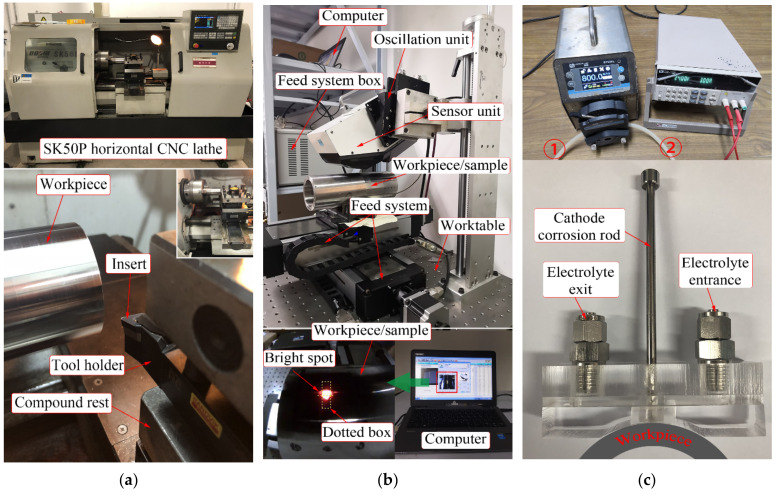
(**a**) The turned experimental details, (**b**) the approach of residual stress measurement by X-ray diffractometer, and (**c**) the electrolytic corrosion device.

Two experiments, as shown in Table 3, were carried out to verify the simulation parameters for turning Inconel 718 pipes (outer diameter × length × wall thickness: 76 × 200 × 8 mm). The Inconel 718 pipes experienced the heat treatment, were annealed, warmed at 720 °C for 8 h, cooled down to 620 °C at 50 °C/h, held at 620 °C for 8 h and quick cooled, and kept at 43 HRC. The main elements and weight of Inconel 718 pipes used in experiments are shown in Table 4. Then, the SK50P horizontal CNC lathe was applied to the turning experiments, as shown in Figure 2a. The workpiece rotates and the compound rest feeds with the tool. The tool holder provided by Sandivk company is DDHNR 2525M 1504. The insert (DNMG 15 04 12-SMR 1105, M-Grade) with physical vapor deposition 0.002 mm TiAlN remains at a 55° top angle and a 1.1906 mm tool nose radius. The other tool geometrical parameters in experiments are displayed in Table 2, consistent with simulations. Moreover, the new sharp insert was used in each turning test.

In this study, an X-ray diffractometer (μ—360n type provided by Pulstec Industrial Co., Ltd., Hamamatsu, Japan) was introduced to acquire residual stress, as displayed in Figure 2b. The X-ray approach is able to assess the residual stress by measuring the lattice character without destroying the material. The Cr K-Beta tube in the X-ray diffractometer generated an X-ray (wavelength 2.08480 Å), and the voltage was maintained at 30 kV and the current at 1.2 mA. In the process of measuring residual stress, the workpiece was placed on the feed system worktable after adjusting the height of the sensor unit with the lead screw. The feed system control box was applied to make the X-ray emitted by the sensor unit coincide with the marked point on the workpiece surface. When the bright spot is in the middle of the dotted box in the computer, the measurement can be started. The oscillation unit continuously adjusted the angle between the X-ray and workpiece surface so that X-rays were irradiated to the 311-crystal plane at a diffraction angle of 150.876° (2-theta) and a diffraction lattice angle of 29.124°. In this manner, the sensor unit measured the lattice character, and the residual stress was calculated using the cosα method by the sensor unit and the computer. The principle of the X-ray residual stress measurement by the cosα method can be found in [11].

In addition, the electrolytic etching method was adopted to strip layers so as to obtain the tendency of residual stress along the negative Y-axis in the turned shallow surface in the experiments, without introducing new stress. When the electrolytic etching was carried out, the electrolytic corrosion device was attached to the cut surface of the workpiece, as shown in Figure 2c. The cathode corrosion rod and the workpiece as an anode were linked to the negative terminal and positive terminal belonging to the itech DC power supply, separately. The electrolyte was 10% NaCl aqueous solution. Moreover, the voltage of 24 V and current of 3 A were maintained during electrolysis. The electrolyte flowed from ① to ② (in Figure 2c) at a speed of 80 mL/min driven by the peristaltic pump, then entered the electrolytic corrosion device from the electrolyte entrance, and flowed through the workpiece surface, and eventually entered the electrolytic cell from the electrolyte exit. The whole workpiece and electrolytic corrosion device were placed in the electrolytic cell. The electrolysis polishing rate was kept at 0.005 mm/s.

### 2.3. FEM Validation

**Figure 3:**
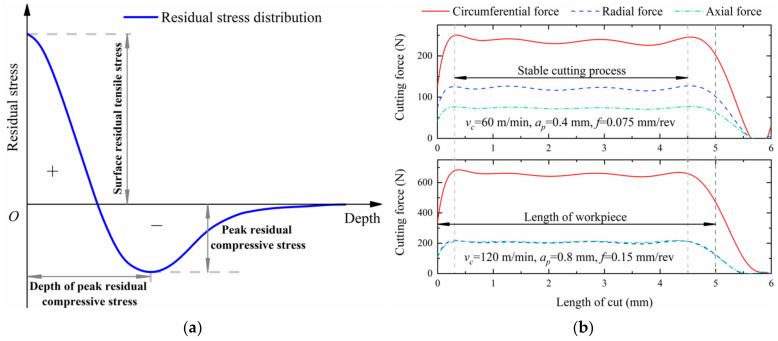
(**a**) The hook-shaped tendency of residual stress in the shallow surface [26]. (**b**) The cutting forces in the turning simulations.

The distribution trend of residual stress along the negative Y-axis in the turned shallow surface is approximately hook-shaped for Inconel 718 material [26], as exhibited in Figure 3a. The cutting process is coupled with thermal-mechanical loads. The thermal load is conductive to be tensile and the mechanical load tends to be compressive for the residual stress [10]. Moreover, the affection of thermal load on residual stresses near the surface is higher than that of mechanical load, but it is opposite to the subsurface. Therefore, the residual stress near the shallow surface of the workpiece gradually falls off from tensile to compressive stress, then increases and approaches a constant value along the depth direction [27]. The cutting forces during the turning tend to be stable within the regions of about 0.5~4.5 mm, as shown in Figure 3b, including circumferential force (F_c_) whose direction is collinear with ***v_c_***, radial force (F_r_) whose direction is consistent with ***a_p_***, and whose direction is axial force (F_a_) consistent with ***f***. Therefore, the residual stress was extracted in the steady cutting. The extraction method of residual stress has been presented in previous research [19,28].

**Figure 4:**
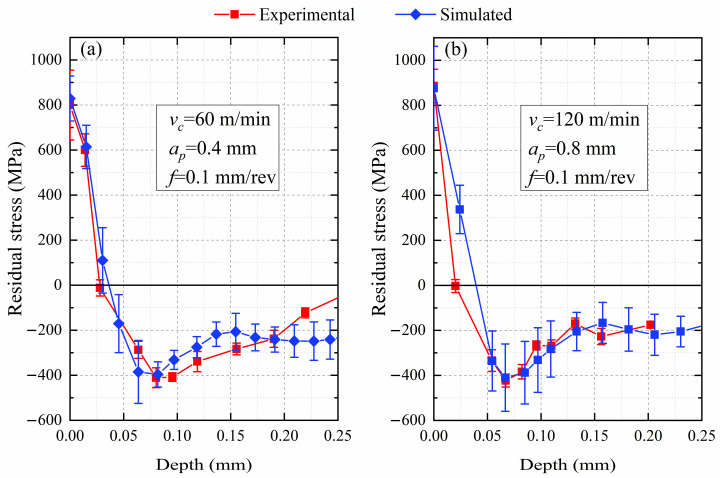
The comparison of the residual stresses in the experiments and simulations, where ***f*** equals 0.1 mm/rev. (**a**) ***v_c_*** and ***a_p_*** equal 60 m/min and 0.4 mm, respectively. (**b**) ***v_c_*** and ***a_p_*** equal 120 m/min and 0.8 mm, respectively [28].

The residual stresses extracted from simulations and measured in experiments are shown in Figure 4, both presenting hook-shaped distributions. The residual stresses near the turned shallow surface are tensile, the direction of residual stresses becomes compressive with the increase of depth, and the compressive stresses climb slowly after reaching extreme values. The simulated residual stresses are slightly higher than those measured in the experiments before the depth of 0.05 mm because the actual electrolytic depth caused by the cylindrical surface of the workpiece is slightly deeper than the product of electrolysis polishing rate and electrolytic time. Moreover, the relative error of the surface residual stresses between the experiment and the simulation is less than 5%, the relative error of the peak compressive residual stresses is within 5%, and the relative error of the depths of compressive stress extreme values below the machined surface is also not beyond 5%, as shown in Figure 4.

**Figure 5:**
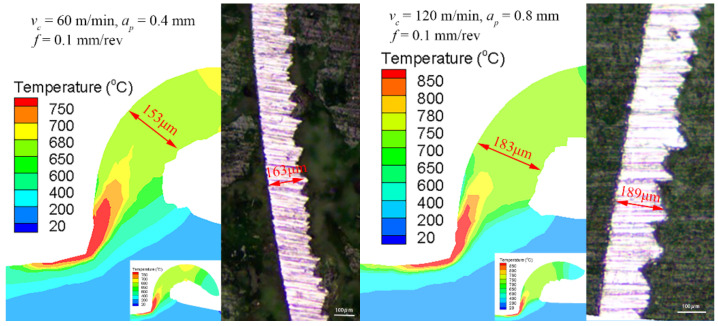
The comparison of the chip thickness in the experiments and simulations [28].

Figure 5 exhibits the chip morphology in the simulations and turning experiments under two groups of cutting parameters. Furthermore, the heat in the cutting process is mainly concentrated in the contact areas between the rake face and the chips (the friction among the rake face and the chips along the secondary zone), and between the flank face and the turned surface of the workpiece (the friction of the tertiary zone). Besides, the heat includes the turned temperature resulting from deformation between the tool nose and the workpiece along the primary zone or shear plane. The error of the chip thickness in the two groups of parameters is 6.1% (***v_c_***, ***a_p_***, and ***f*** equal 60 m/min, 0.4 mm, and 0.1 mm/rev, respectively) and 3.2% (***v_c_***, ***a_p_***, and ***f*** equal 120 m/min, 0.8 mm, and 0.1 mm/rev, respectively), as shown in Figure 5. In summary, the simulation parameter setting is effective, comparing the distribution of residual stresses, including maximum tensile residual stress on turned surface and compressive stress extreme values, and chips’ thickness in simulations and experiments. Therefore, the following research was further carried out.

### 2.4. Simulation Planning

The analysis of Section 2.3 shows that the parameter setting of Section 2.1 is reasonable. Therefore, the surface residual stress after turning Inconel 718 material was studied in the current work on the basis of the small feed rate as well as the small cutting depth. The simulation planning was designed as in Table 5, containing 24 (4 × 3 × 2) sets of parameters. The simulations were performed on a computer with two Intel Xeon E5 2.3 GHz processors. The cutting forces, cutting temperatures, and surface residual stresses extracted from 24 simulations are analyzed in Section 3.

## 3. Results and Discussion

### 3.1. Analysis of Cutting Forces

**Figure 6:**
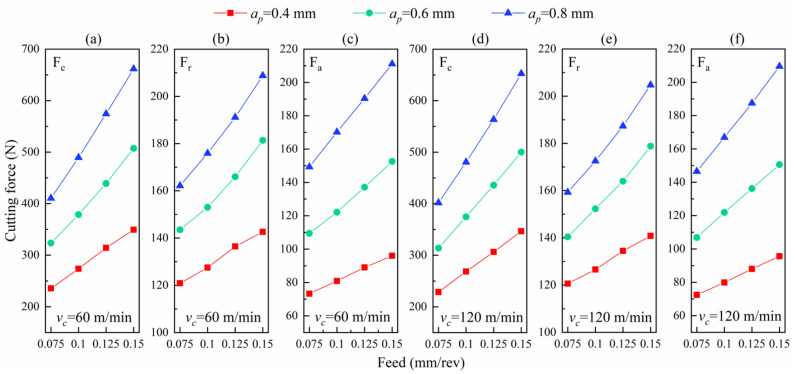
The trends of the cutting force components with feed at each cutting speed. (**a**) The circumferential force (60 m/min), (**b**) the radial force (60 m/min), and (**c**) the axial force (60 m/min). (**d**) The circumferential force (120 m/min), (**e**) the radial force (120 m/min), and (**f**) the axial force (120 m/min).

**Figure A1:**
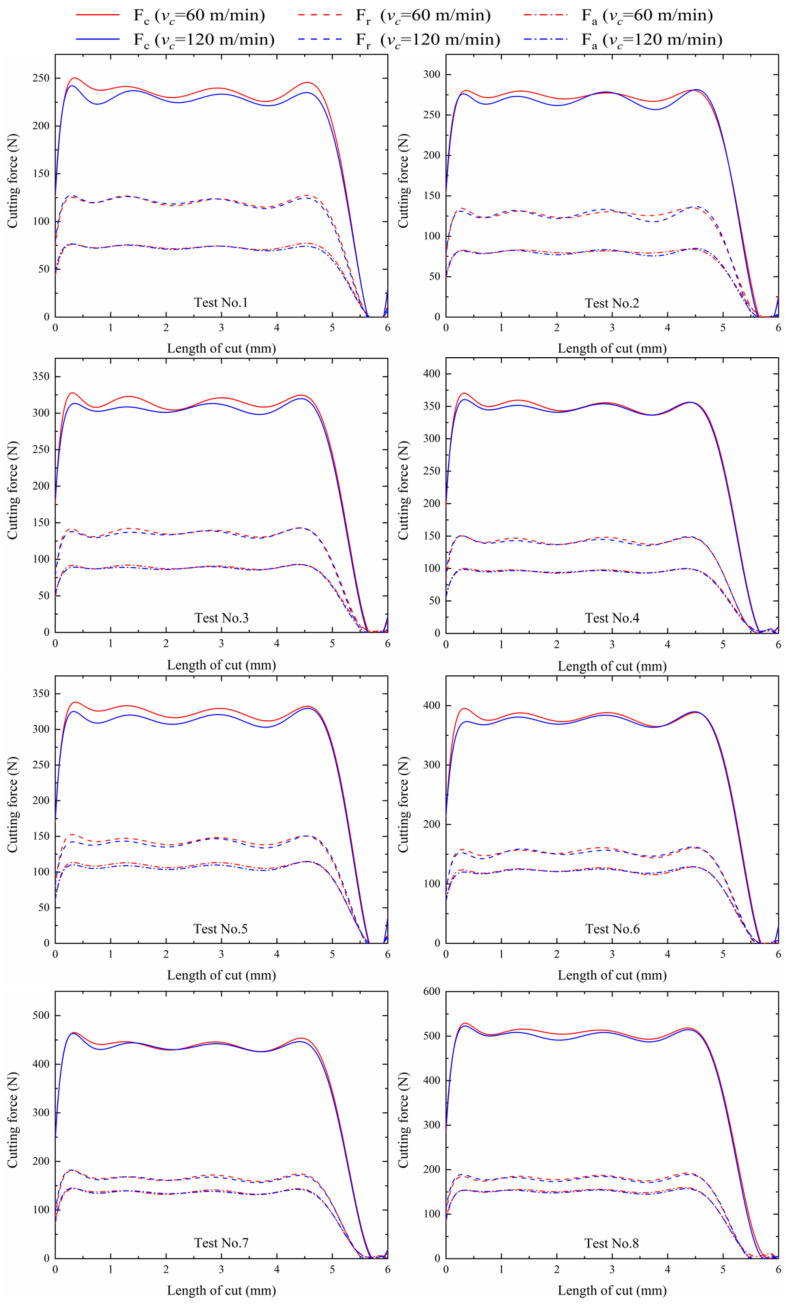

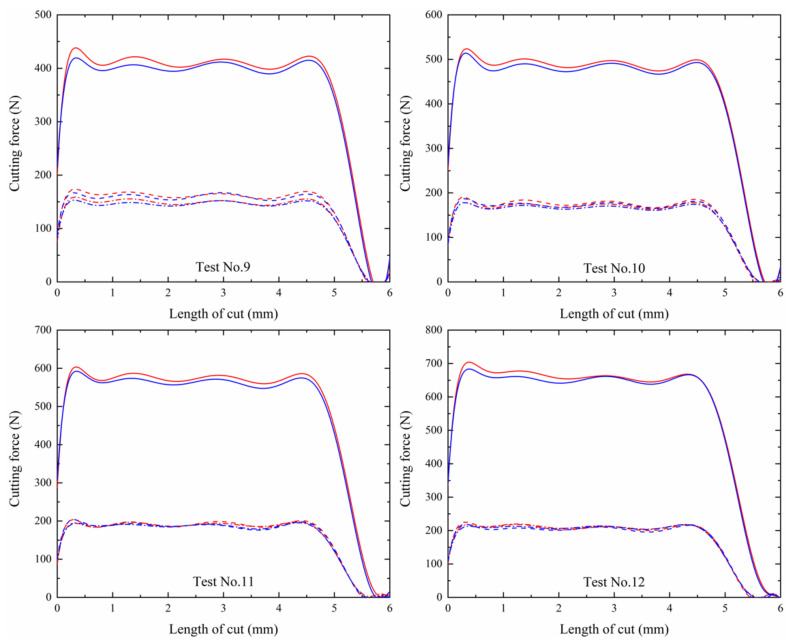
Ten-order polynomial fitting diagram of cutting forces in 24 simulations.

The cutting forces extracted in simulations consist of three parts: the circumferential force (F_c_), the radial force (F_r_), and the axial force (F_a_), as shown in Appendix A Figure A1. The changes of cutting forces with feed rate are shown in Figure 6 in the process of the cutting simulation. Figure 6a reveals that the circumferential force (F_c_) rises with the growing feed rate (in the region of 0.075~0.15 mm/rev) when cutting speed remains at 60 m/min. Meanwhile, F_c_ arises with the increasing depth of cut, because the larger the cutting depth is, the more obvious the slope is. Similarly, the radial force (F_r_) and the axial force (F_a_) also rise with the growth of the feed rate, and their changes are more obvious with the rise of the feed rate when the depth of the cut goes up, as shown in Figure 6b,c. The circumferential, radial, and axial forces also show the same trends at the high cutting speed in Figure 6d–f.

**Figure 7:**
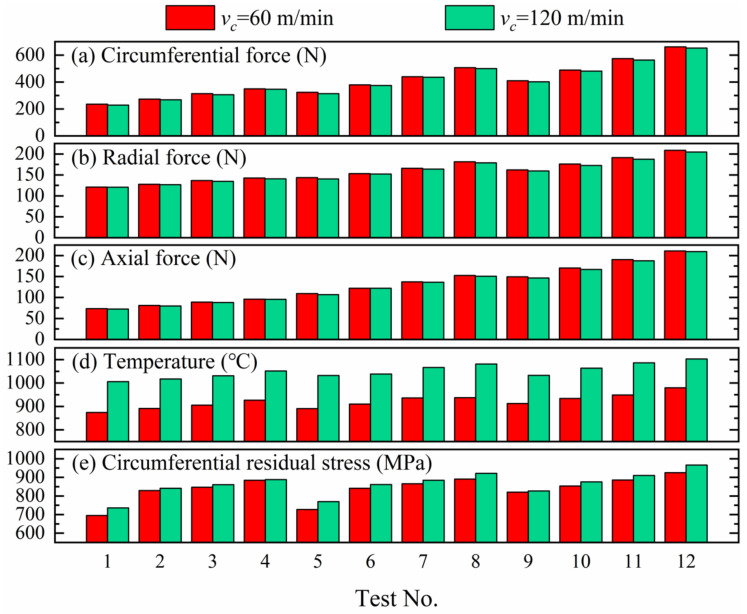
(**a**) The comparison of the circumferential forces at 60 and 120 m/min. (**b**) The comparison of the radial forces at 60 and 120 m/min. (**c**) The comparison of the axial forces at 60 and 120 m/min. (**d**) The comparison of the cutting temperatures at 60 and 120 m/min. (**e**) The comparison of the circumferential residual stress at 60 and 120 m/min.

The circumferential cutting forces at low speed (60 m/min) are higher than those at high speed (120 m/min), as seen from Figure 7a, which is also reflected in the radial component of the cutting forces in Figure 7b, as well as the axial component in Figure 7c. The reason for these is that the change of cutting forces with cutting speed is closely related to the cutting temperature. The cutting temperatures at high speed are higher than those at low speed, which causes a more intense thermal softening effect. Thus, the cutting forces are lower at the high than the low cutting speed.

### 3.2. Analysis of Cutting Temperatures

**Figure 8:**
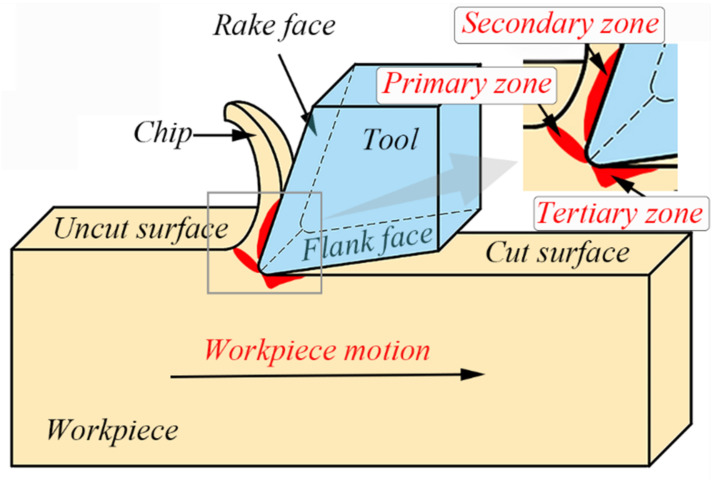
The schematic diagram of the cutting process and three shear zones [5].

**Figure 9:**
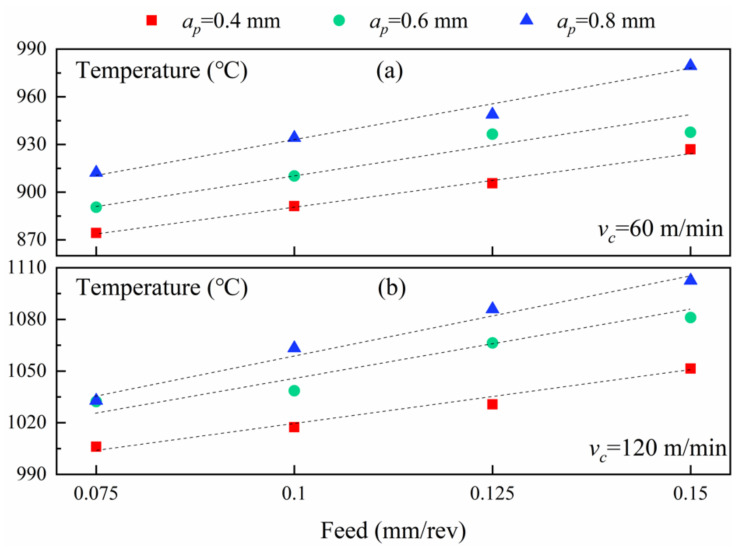
(**a**) The variations of the cutting temperatures as feeds and cutting depths at 60 m/min. (**b**) The variations of the cutting temperature as feeds and cutting depths at 120 m/min.

**Figure A2:**
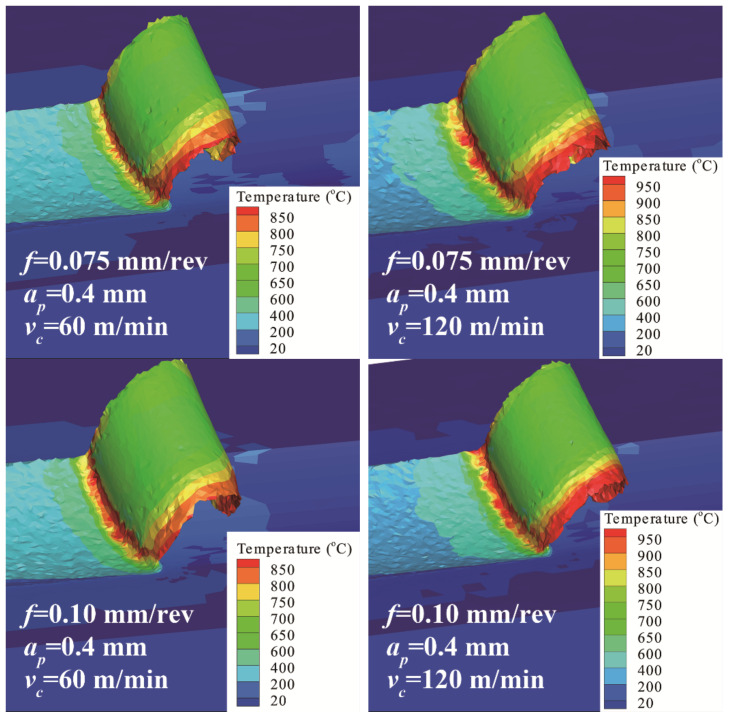

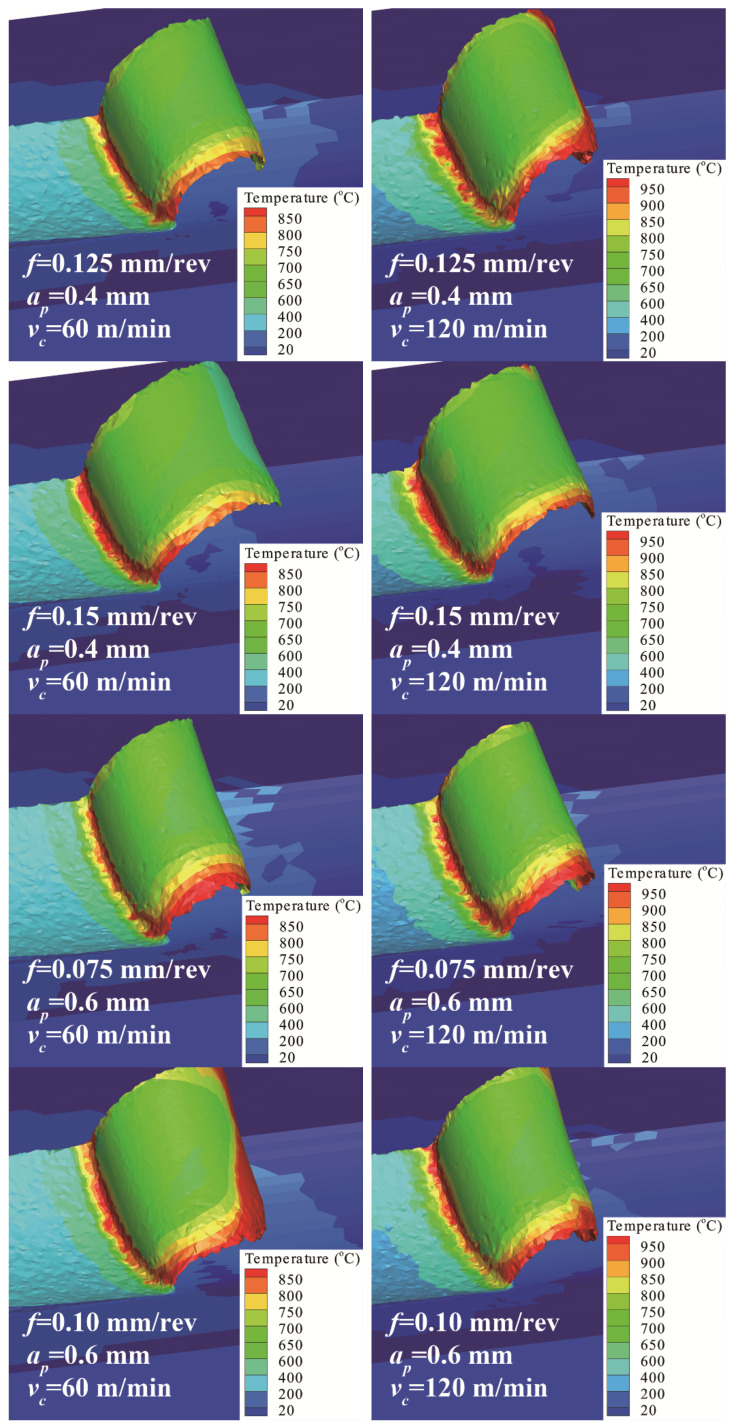

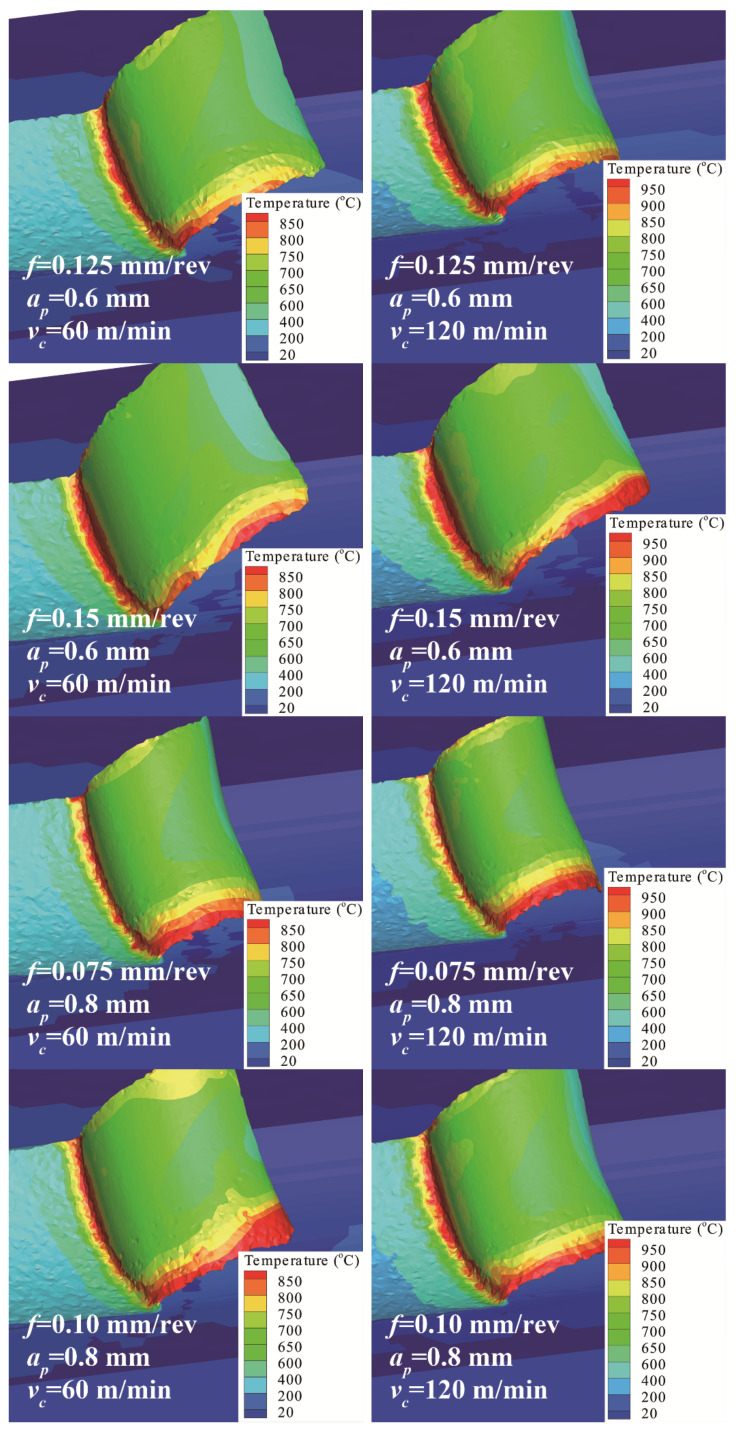

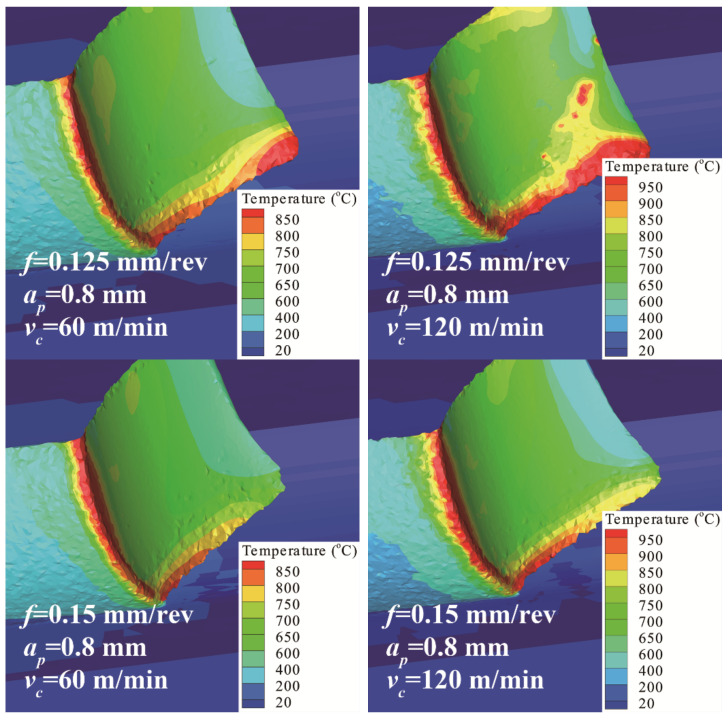
The temperatures of tool nose contact zone in the cutting process in the simulations.

The tool extrudes material on the front of the tool nose for turning. As the extrusion process continues, the material on the front of the tool nose slides along the shear plane, undergoes plastic deformation, and accumulates continuously to form chips. Meanwhile, the friction at contact of the chips and insert, and the friction among the insert and workpiece, also accompany this process. As shown in Figure 8, the material in the primary zone produces plastic deformation, which induces the temperature increase in the cutting region. Moreover, the friction among the rake face and chips in the secondary zone also raises the temperature of the cutting region. The increasing temperature further arises from the friction generated by the machined workpiece surface and tool flank face in the tertiary zone. The tool nose temperatures in the cutting process in the simulations are shown in Appendix A Figure A2. Figure 9a exhibits the changes of cutting temperature with raising the feed rate under the varying depth of cut at the speed of 60 m/min. The temperature in the cutting area climbs slowly with the feed rate for each depth of cut (the dotted lines in Figure 9 are not fitting lines, only changing trends). Of course, the temperature also decreases slightly for the 0.6 mm cutting depth and 0.15 mm/rev feed. Besides, the temperature in the cutting area rises with the growing cutting depth, which is correlated with degree of extrusion and sliding deformation in shear regions. Figure 9b displays the relationship between cutting temperature and feed when the cutting speed is 120 m/min. Generally, the cutting temperature climbs slowly with the rising feed rate. Moreover, the cutting temperature at 120 m/min grows with the rising depth of cut, which is parallel to that at 60 m/min.

The temperatures of material in the contact region of the tool nose during turning are high at 120 m/min, compared to significantly lower temperatures at 60 m/min, as displayed in Figure 7d. The frictions in the secondary zone and tertiary zone are further intensified when the cutting speed increases. Therefore, more heat is accumulated in the material at the front of the tool nose at high turning speed. The cutting temperature is higher at high cutting speed than the low speed for the same heat dissipation conditions.

### 3.3. Analysis of Surface Residual Stress

**Figure A3:**
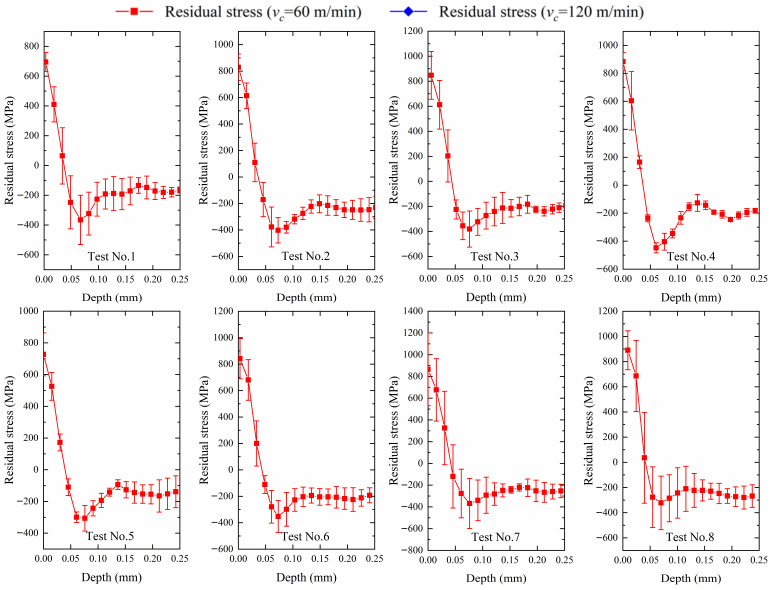

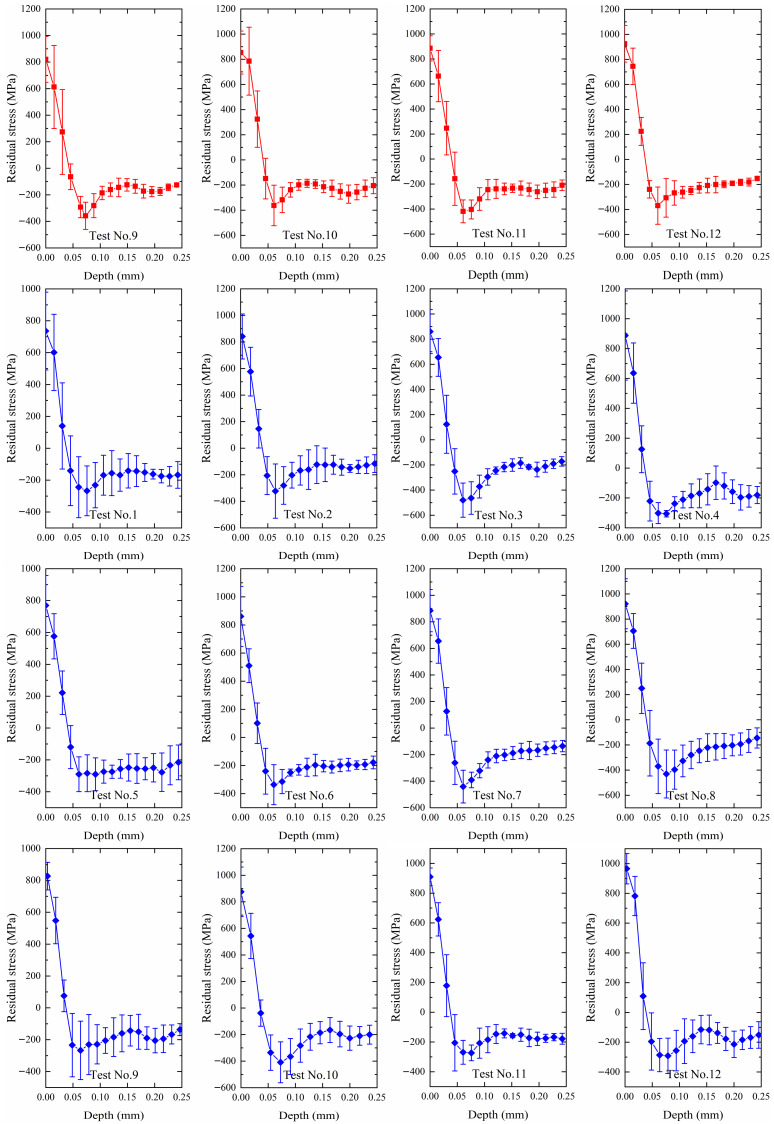
The residual stresses’ distributions extracted in 24 simulations.

The thermal effect is beneficial for generating tensile residual stress, while the mechanical load tends to be compressive [10]. The surface residual stress on the workpiece arises from the coupling of the thermal-mechanical load. Therefore, the tensile residual stress was generated on the shallow surface region of material for turning Inconel 718 pipes in the present study, which showed that the thermal load had a more obvious effect on the formation of residual stress near the shallow surface region than the mechanical load. Further, the residual stresses’ distributions extracted in the simulations are shown in Appendix A Figure A3. The surface circumferential residual stresses climb with rising feed rate for each cutting depth at 60 m/min, as illustrated in Figure 7e. Besides, when the depth of cut is 0.4 or 0.6 mm, the residual stress rises more for 0.075~0.10 mm/rev than other feed rates. The residual stress rises more evenly with the growing feed for an 0.8 mm cutting depth. The same trends were also shown in [11,29] concerning the variation of residual stress with feed rate. In a similar manner, the surface residual stresses have similar trends with the change of feed at 120 m/min, but the amplitudes of residual stresses are slightly higher than that at 60 m/min because the thermal effect is more significant at high than at low speed. The relationship between residual stress and cutting speed is consistent with that in [30].

## 4. Establishment of Prediction Model

### 4.1. Introduction of Cutting Tool Angles

**Figure 10:**
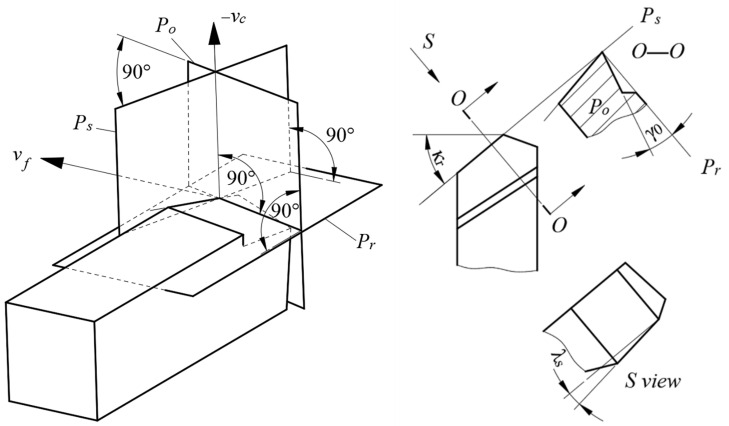
The orthogonal plane coordinate system and the tool parameters [31].

The tool parameters are determined by the reference plane used in the manufacturing process of the tool, as shown in Figure 10. *P_r_* is the base plane and perpendicular to −***v_c_*** axis, *P_s_* is the cutting plane, and *P_o_* is the orthogonal plane. Three planes, *P_r_*, *P_s_*, and *P_o_*, are perpendicular to each other and intersect at a point on the cutting edge. Moreover, the plane *P_s_* is tangent to the cutting edge. Figure 10 shows the position relationships between *P_r_*, *P_s_*, and *P_o_*. ***v_f_*** is the axis of feed motion and ***v_c_*** is the axis of cutting motion. In this way, the rake angle ***γ*_0_** (the O–O view), the tool cutting-edge angle ***κ_r_*** (the top view), and the inclination angle ***λ_s_*** (S-direction view) are determined.

**Figure 11:**
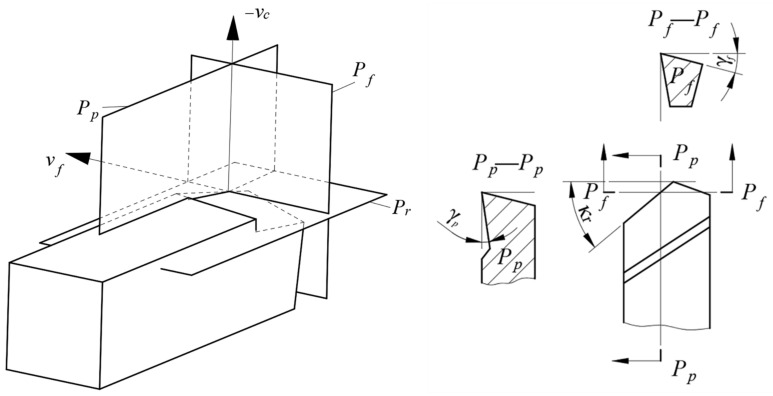
The assumed working plane coordinate system and the tool parameters [31].

The reference plane is determined according to the turning movement direction, as shown in Figure 11. *P_r_* is the base plane, which is determined by the feed direction of the tool and the direction of depth of cut, *P_f_* is the working plane in the turning process and is composed of the feed direction and the cutting speed direction, and *P_p_* is the back plane, which is perpendicular to the base plane and the working plane in the cutting process. Further, three planes, *P_r_*, *P_f_*, and *P_p_*, are perpendicular to each other at a point on the cutting edge. After the two planes (*P_p_* and *P_f_*) are determined, the back-rake angle of the tool ***γ_p_*** in the *P_p_* plane and the side-rake angle ***γ_f_*** in the *P_f_* plane can be determined, as shown in *P_p_*–*P_p_* view and *P_f_*–*P_f_* view.

Therefore, the conversion relationships of ***γ_p_*** (back-rake angle), ***γ_f_*** (side-rake angle), and tool parameters (the tool cutting-edge angle ***κ_r_***, the rake angle ***γ*_0_**, and the inclination angle ***λ_s_***) during the tool design and manufacturing process are shown in Equations (2) and (3), respectively [31]:

(2) $$\tan\gamma_{p}=\tan\gamma_{0}\cos\kappa_{r}+\tan\lambda_{s}\sin\kappa_{r}$$

(3) $$\tan\gamma_{f}=\tan\gamma_{0}\sin\kappa_{r}-\tan\lambda_{s}\cos\kappa_{r}$$

### 4.2. Prediction Model and Determination of Parameters

**Figure 12:**
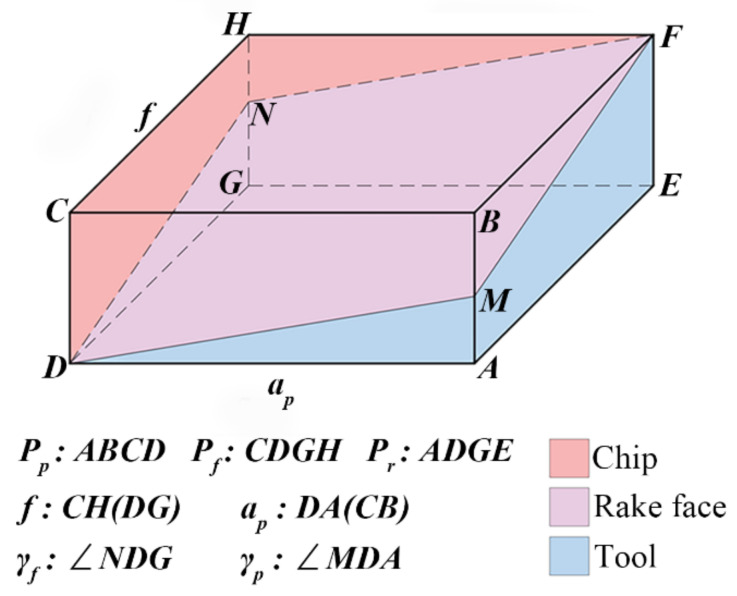
The diagram of the simplified tool-chip model.

The relationship between the insert and the chip was simplified as shown in Figure 12. The point D is the selected point on the cutting edge, and the planes ABCD, CDGH, and ADGE are the back plane (*P_p_*), the working plane (*P_f_*), and the base plane (*P_r_*), respectively. Thus, the angle ***γ_p_*** is the ∠MDA and the angle ***γ_f_*** is the ∠NDG. Moreover, the area of the plane ADGE, which is the projection plane of the contact surface among tool rake face and chips onto the base plane, is equal to *f* × *a_p_*; besides, the projection areas of the contact surface among tool rake face and chips onto the working plane and the back plane are obtained as |*f* × *a_p_* × tan*γ_p_*| and |*a_p_* × *f* × tan*γ_f_*|, separately. The three areas are represented by S1, S2, and S3, as follows in Equation (4):

(4) $$\left\{\begin{array}{l} S_{1}=f\times a_{p},\\ S_{2}=\left|f\times a_{p}\times \tan\gamma_{p}\right|=f\times a_{p}\times \left|\tan\gamma_{p}\right|,\\ S_{3}=\left|a_{p}\times f\times \tan\gamma_{f}\right|=a_{p}\times f\times \left|\tan\gamma_{f}\right|. \end{array}\right.$$

The surface residual stress on the workpiece after turning results from the thermal-mechanical load during machining. Therefore, the surface residual stress ($\sigma_{surface}$) was recorded as a function (Equation (5)) of the cutting temperature (*T*) and the cutting force (*F*) in the present study:

(5) $$\sigma_{surface}=f\left(T,F\right)$$

The relationships of cutting force with feed and cutting depth were analyzed in Section 3.1. The cutting force rose with the increasing feed and cutting depth. Therefore, the circumferential, radial, and axial stresses of the cutting forces acting on the contact region of the material and insert were expressed as in Equation (6):

(6) $$\sigma_{c}=\frac{F_{c}}{S_{1}},\;\sigma_{r}=\frac{F_{r}}{S_{2}},\;\sigma_{a}=\frac{F_{a}}{S_{3}}.$$

where $\sigma_{c}$ denotes the stress component caused by $F_{c}$ applying to $S_{1}$, $\sigma_{r}$ denotes the stress component caused by $F_{r}$ applying to $S_{2}$, and $\sigma_{a}$ denotes the stress component caused by $F_{a}$ applying to $S_{3}$.

The cutting speed had a crucial influence on the cutting temperature based on the analysis in Section 3.2. The rise of cutting speed causes growing cutting temperature. Therefore, when expressing the influence of cutting temperature on surface residual stress, the cutting speed was introduced into the temperature term, as in Equation (7):

(7) $$\sigma_{T}=g\left(T,v_{c}\right)$$

In which $\sigma_{T}$ represents the stress component relevant to the thermal effect.

Equation (5) can be further rewritten as:

(8) $$\sigma_{surface}=f\left(\sigma_{T},\sigma_{c},\sigma_{r},\sigma_{a}\right)$$

The surface residual stress caused by thermal-mechanical load in turning can be expressed in two parts: (1) including cutting temperature and cutting speed ($\sigma_{part\;1}$), and (2) including cutting force, feed, cutting depth, and tool angle ($\sigma_{part\;2}$). Moreover, a concrete function expression was provided as in Equation (9):

(9) $$\left\{\begin{array}{l} \sigma_{part\;1}=B_{0}\frac{T^{m_{1}}}{v_{c}^{m_{2}}},\\ \sigma_{part\;2}=-B_{1}{\left(\frac{F_{c}}{a_{p}^{n_{1}}f}\right)}^{n_{2}}-B_{2}{\left(\frac{F_{r}}{fa_{p}^{n_{3}}\left|\tan\gamma_{p}\right|}\right)}^{n_{4}}-B_{3}{\left(\frac{F_{a}}{a_{p}^{n_{5}}f\left|\tan\gamma_{f}\right|}\right)}^{n_{6}}. \end{array}\right.$$

where $B_{0}$ is the coefficient of temperature and cutting speed term, $B_{1}∼B_{3}$ are the coefficients of cutting forces, cutting, and tool parameters terms, $m_{1}$ and $m_{2}$ are the exponents of temperature and cutting speed term, and $n_{1}∼n_{6}$ are the exponents of cutting forces, cutting, and tool parameters terms.

The functional relationship, shown in Equation (10), about surface residual stress after turning, cutting temperature, cutting force, cutting parameters, and tool parameters was established based on the above discussion.

(10) $$\sigma_{surface}=\sigma_{part\;1}+\sigma_{part\;2}=B_{0}\frac{T^{m_{1}}}{v_{c}^{m_{2}}}-B_{1}{\left(\frac{F_{c}}{a_{p}^{n_{1}}f}\right)}^{n_{2}}-B_{2}{\left(\frac{F_{r}}{fa_{p}^{n_{3}}\left|\tan\gamma_{p}\right|}\right)}^{n_{4}}-B_{3}{\left(\frac{F_{a}}{a_{p}^{n_{5}}f\left|\tan\gamma_{f}\right|}\right)}^{n_{6}}$$

The thermal effect results in the tensile residual stress and the mechanical load leads to the compressive residual stress. The positive value is utilized to express the tensile residual stress and the negative value represents the compressive residual stress. Therefore, in Equation (10), the thermal effect term was represented by a positive sign (omitted in Equation (10)) and the mechanical load terms used a negative sign. Then, the genetic algorithm toolbox in MATLAB was applied to obtain the undetermined parameters in Equation (10). The optimization function is shown as Equation (11), which minimizes the sum of the absolute values of the differences between the predicted and the original values:

(11) $$f_{GA}=\min\left[\sum_{i=1}^{N}\left|B_{0}\frac{T{\left(i\right)}^{m_{1}}}{v_{c}{\left(i\right)}^{m_{2}}}-B_{1}{\left(\frac{F_{c}\left(i\right)}{a_{p}{\left(i\right)}^{n_{1}}f\left(i\right)}\right)}^{n_{2}}\right.\right.-B_{2}{\left(\frac{F_{r}\left(i\right)}{f\left(i\right)a_{p}{\left(i\right)}^{n_{3}}\left|\tan\gamma_{p}\right|}\right)}^{n_{4}}\left.\left.-B_{3}{\left(\frac{F_{a}\left(i\right)}{a_{p}{\left(i\right)}^{n_{5}}f\left(i\right)\left|\tan\gamma_{f}\right|}\right)}^{n_{6}}-\sigma_{sim}\left(i\right)\right|\right]$$

where $f_{GA}$ is the optimization objective, *N* is the total of simulations (N=24), *i* denotes the ith simulation (i=1,2,⋯,N.), and $\sigma_{sim}\left(i\right)$ denotes the ith simulated residual stress on the turned surface.

In the genetic algorithm toolbox, the population size was set as 20,000, and the stopping criteria contained the generations and the function tolerance, which were set as 12,000 and 10^−200^, separately. Considering that the tensile residual stress is positive, and the compressive residual stress is negative, the lower limit of the undetermined parameters in Equation (11) was kept as 0. After 1424 iterations, the function tolerance was achieved and the value of $f_{GA}$ stopped at 319.032. The undetermined parameters are shown in Table 6.

**Figure 13:**
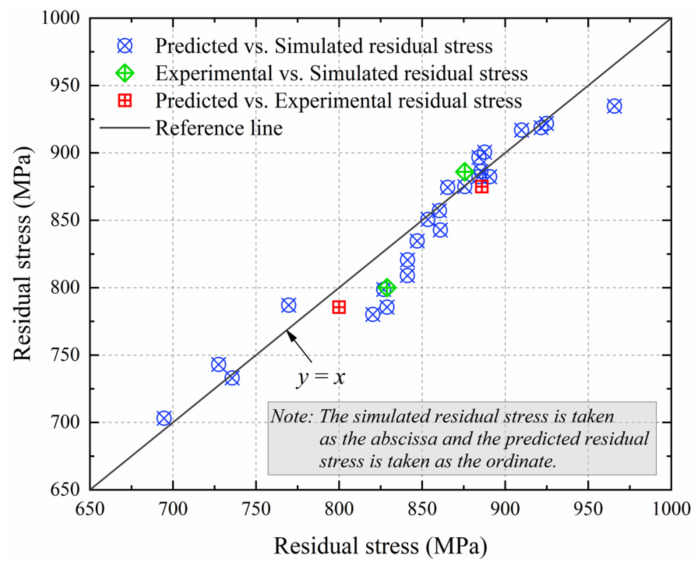
The predicted, simulated, and experimental residual stresses.

As shown in Figure 13, the stress points are distributed near the reference line (y = x) when the simulated residual stresses are taken as the abscissa and the predicted residual stresses are taken as the ordinate. Meanwhile, stress points are composed of the experimental and the simulated values, and the predicted and the experimental values are also distributed near the reference line (y = x). Equation (12) was applied to calculate the correlation coefficient (R) of the predicted and simulated residual stresses, and Equation (13) was applied to calculate the absolute average error (AARE) of the predicted and simulated residual stresses [32]. Therefore, R is 0.9624 and AARE is 13.40%, which further shows that Equation (10) is accurate to predict the turned surface residual stress for Inconel 718 within the feed ranges of 0.075~0.15 mm/rev.

(12) $$R=\frac{\sum_{i=1}^{N}\left(\sigma_{sim}\left(i\right)-{\bar{\sigma }}_{sim}\right)\left(\sigma_{pre}\left(i\right)-{\bar{\sigma }}_{pre}\right)}{\sqrt{\sum_{i=1}^{N}{\left(\sigma_{sim}\left(i\right)-{\bar{\sigma }}_{sim}\right)}^{2}}\sqrt{\sum_{i=1}^{N}{\left(\sigma_{pre}\left(i\right)-{\bar{\sigma }}_{pre}\right)}^{2}}}$$

(13) $$AARE=\frac{1}{N}\sum_{i=1}^{N}\left|\frac{\sigma_{sim}\left(i\right)-\sigma_{pre}\left(i\right)}{\sigma_{sim}\left(i\right)}\right|\times 100\%$$

where *N* equals 24, ${\bar{\sigma }}_{sim}$ is the mean of all 24 simulated residual stresses on the turned surface, $\sigma_{pre}\left(i\right)$ is the predicted residual stress for the ith simulation, and ${\bar{\sigma }}_{pre}$ is the mean of all 24 predicted residual stresses.

### 4.3. How to Use the Model

In the present work, the cutting temperature and the cutting forces were extracted in the simulations. However, in the production, the cutting temperature and the cutting forces can be separately obtained by the infrared camera and the force sensor, and the residual stresses on the turned surface can be measured by the X-ray diffractometer. Then, combined with cutting parameters, the coefficients of Equation (10) can be acquired by utilizing the genetic algorithm so as to accomplish the prediction of the surface residual stress in the turning process, which can guide the process planning. When using the prediction model, tan***γ_p_*** and tan***γ_f_*** cannot be equal to zero because they are the denominators in Equation (10). Thus, there are the following restrictions for ***κ_r_***, ***γ*_0_**, and ***λ_s_***:

1. ***γ*_0_** and ***λ_s_*** cannot be zero at the same time.
2. When ***κ_r_*** equals 45° or 135°, |***γ*_0_**| ≠ |***λ_s_***|.

## 5. Conclusions

The turned surface residual stress for Inconel 718 material was studied in the present work. The novel surface residual stress prediction equation was proposed on the basis of cutting force, cutting temperature, feed, depth of cut, and cutting speed in the turning process. The novelty of this model is that the tool angles containing the rake angle, the tool cutting-edge angle, and the inclination angle were introduced in a turned surface residual stress prediction equation for Inconel 718 for the first time. The main research conclusions are summed up as follows:

1. The distributions of residual stress in two experiments of turning Inconel 718 were consistent with that in the simulations. Furthermore, under the 24 parameters studied in the simulations, the circumferential cutting force, the radial cutting force, and the axial cutting force rose with the growing feed, no matter at high speed or low speed. With the increasing cutting depth, cutting forces rose more obviously. The cutting forces were lower at high cutting speed than at low cutting speed. The cutting temperature had an upward trend with the growth of the feed rate. The cutting temperature at high speed was slightly higher than that at low speed. The circumferential residual stress climbed with the rising feed rate within 0.075~0.15 mm/rev.
2. A novel empirical residual stress prediction equation was proposed. In this equation, ***κ_r_***, ***γ*_0_**, and ***λ_s_*** were introduced for the first time. Further, the optimization objective function was established according to the residual stress prediction equation, and the undetermined coefficients in the equation were obtained by using the genetic algorithm in MATLAB.
3. Comparing the predicted, the simulated, and the measured stress, the results show that this prediction equation is accurate in predicting turned surface residual stress for Inconel 718 material within the feed rate of 0.075~0.15 mm/rev. The R value between the predicted and simulated stress was 0.9624 using the Pearson correlation analysis, and the average absolute error (AARE) was 13.40%, which further shows the accuracy of the prediction equation.
4. When Equation (10) was applied, there were two restrictions: the rake angle and the inclination angle cannot be zero at the same time, and |***γ*_0_**| cannot equal |***λ_s_***| when ***κ_r_*** is 45° or 135°. Therefore, future studies can be carried out based on the present work.

In practical application, it is necessary to measure the residual stress on turned surfaces during turning of parts for the purpose of judging the surface quality of parts. The prediction model proposed in this paper can replace this process and improve the production efficiency. In addition, this model can be used in the real-time monitoring of turning Inconel 718, and can also predict the residual stress level so as to provide guidance for the process planning.

## Figures and Tables

**Table 1:** The material properties of the Inconel 718 used in the simulations [25].

Thermal Conductivity (W/(m·K))	Specific Heat (J/(kg·K))	Thermal Expansion Coefficient (10^−6^/K)	Melting Temperature (K)
10.53 (293 K)	435 (293 K)	11.8 (293–373 K)	1573
14.7 (373 K)	481.4 (573 K)	13 (293–573 K)	
17.8 (573 K)	514.8 (773 K)	14.1 (293–673 K)	
19.6 (773 K)	573.4 (973 K)	14.8 (573–873 K)	

**Table 2:** The tool geometry used in turning simulations and experiments.

Tool Parameters	Values
Rake angle (degree)	−6
Relief angle (degree)	6
Lead angle (degree)	−17.5
Inclination angle (degree)	−7
Coating (thickness)	TiAlN (0.002 mm)

**Table 3:** The cutting regime in the experiments.

Turning Experiments	Cutting Speed (m/min)	Depth of Cut (mm)	Feed Rate (mm/rev)
1	60	0.4	0.1
2	120	0.8	

**Table 4:** The main elements and weight of Inconel 718 pipes used in experiments.

Elements	Ni	Fe	Cr	Nb	Mo	Ti
Weight	52.86%	19.15%	19.085%	5.085%	3.105%	0.71%

**Table 5:** The planning of the cutting parameters in the simulations.

Test No.	Feed Rate (mm/rev)	Depth of Cut (mm)	Cutting Speed (m/min)
1	0.075	0.4	60, 120
2	0.10		
3	0.125		
4	0.15		
5	0.075	0.6	
6	0.10		
7	0.125		
8	0.15		
9	0.075	0.8	
10	0.10		
11	0.125		
12	0.15		

**Table 6:** The parameters in the predicted model of the surface residual stress.

Parameters	*B*_0_	*m*_1_	*m*_2_	*B*_1_	*n*_1_	*n*_2_	*B*_2_	*n*_3_	*n*_4_	*B*_3_	*n*_5_	*n*_6_
Value	15.332	0.732	0.118	0.444	0.133	1.096	1.736	0.543	0.555	3.998	2.13	0.289

## Data Availability

The data is contained within this article.

## Nomenclature

*v_c_*	Cutting speed (m/min)	***v_f_***	Direction of feed motion
***a_p_***, DA(CB)	Depth of cut (mm)	A	Yield strength (MPa)
***f***, CH(DG)	Feed rate (mm/rev)	B	Strain hardening coefficient (MPa)
FEM	Finite element model	C	Strain rate hardening coefficient
F	Cutting force (N)	m	Thermal softening exponent
F_c_	Circumferential force (N)	n	Strain hardening exponent
F_r_	Radial force (N)	S_1_	Projection area of chip and rake face contact surfaces on the base plane
F_a_	Axial force (N)	S_2_	Projection area of chip and rake face contact surfaces on the working plane
*P_r_*, ADGE	Base plane	S_3_	Projection area of chip and rake face contact surfaces on the back plane
*P_s_*,	Cutting plane	*T*	Temperature (°C)
*P_o_*,	Orthogonal plane	*T_room_*	Room temperature (°C)
*P_p_*, ABCD	Back plane	*T_melt_*	Melting temperature (°C)
*P_f_*, CDGH	Working plane	*B*_0_	Coefficient of temperature and cutting speed term
3D	Three-dimensional		
***κ_r_***	Tool cutting-edge angle (°)	*B*_1_~*B*_3_	Coefficients of cutting forces, cutting, and tool parameters’ terms
***γ*_0_**	Rake angle (°)	*m*_1_, *m*_2_	Exponents of temperature and cutting speed terms
***λ_s_***	Inclination angle (°)	*n*_1_~*n*_6_	Exponents of cutting forces, cutting, and tool parameters’ terms
***γ_p_***, ∠MDA	Back-rake angle (°)	*N*	Total of simulations (*N* = 24)
***γ_f_***, ∠NDG	Side-rake angle (°)	*i*	The ith simulation (*I* = 1, 2, ⋯, *N*.)
***R***	Correlation coefficient	AARE	Absolute average error
$\bar{\varepsilon }$	Equivalent plastic strain	$\bar{\sigma }$	Equivalent plastic stress (MPa)
$\dot{\bar{\varepsilon }}$	Equivalent plastic strain rate ($s^{-1}$)	${\dot{\bar{\varepsilon }}}_{0}$	Reference equivalent plastic strain rate ($s^{-1}$)
$\sigma_{surface}$	Surface residual stress	$\sigma_{c}$	Stress component caused by F_c_ applying to S_1_
$\sigma_{r}$	Stress component caused by F_r_ applying to S_2_	$\sigma_{a}$	Stress component caused by Fa applying to S_3_
$\sigma_{T}$	Stress component relevant to thermal effect	$\sigma_{part\;1}$	Part of surface residual stress relevant to cutting temperature and cutting speed
${\bar{\sigma }}_{sim}$	Mean of all 24 simulated residual stresses on the surface	$\sigma_{part\;2}$	Part of surface residual stress relevant to cutting force, feed, cutting depth, and tool angle
$\sigma_{sim}\left(i\right)$	The ith simulated residual stress on the surface	$\sigma_{pre}\left(i\right)$	The predicted residual stress on the surface for the ith simulation
$f_{GA}$	Optimization objective	${\bar{\sigma }}_{pre}$	Mean of all 24 predicted residual stresses on the surface
